# An Interesting Case of Moyamoya Disease, a Rare Cause of Transient Ischemic Attacks

**DOI:** 10.7759/cureus.9736

**Published:** 2020-08-14

**Authors:** Amit Sapra, Priyanka Bhandari, Rebecca Dix, Shivani Sharma, Eukesh Ranjit

**Affiliations:** 1 Family Medicine, Southern Illinois University School of Medicine, Springfield, USA

**Keywords:** moyamoya disease, strokes, cerebrovascular accidents, rare disorder, acute cva, intracerebral hemorrhage, brain mri, diagnostic cerebral angiogram, computed tomography angiography, transient ischemic attack (tia)

## Abstract

Moyamoya disease is a rare, chronic, idiopathic progressive disease characterized by irreversible vascular occlusion of the vessels of the Circle of Willis. The disease was initially considered to be limited to the East Asian population, but now the disease is being reported all over the globe in people of multiple ethnicities. It is crucial that clinicians are aware of the disease and its presentation to prevent under-recognition of the condition. We describe the case of a 44-year-old Caucasian female with a history of hypertension, depression, gastroesophageal reflux disease (GERD), and morbid obesity diagnosed with Moyamoya disease after she presented to the emergency department with recurrent stroke-like symptoms.

## Introduction

Moyamoya disease is a manifestation of chronic progressive narrowing of the arteries at the base of the brain. The narrowing can eventually result in stroke due to complete blockage.

As a compensatory mechanism for the narrowing arteries, the brain creates collateral blood vessels to deliver oxygen-rich blood to deprived areas of the brain. The name, moyamoya, means 'puff of smoke' in Japanese and originates from the abnormal vascular network's angiographic appearance at the base of the brain [1]. These tiny moyamoya collaterals are more fragile than normal blood vessels and can rupture and bleed into the brain, causing hemorrhages.

Our patient is a 44-year-old Caucasian female who presented with symptoms suggestive of recurrent transient ischemic attacks (TIA), which were later found to be due to Moyamoya disease after extensive investigation.

## Case presentation

Our patient is a 44-year-old Caucasian female with a history of essential hypertension, depression, gastroesophageal reflux disease (GERD), and morbid obesity who presented with a 10-day history of fluctuating left upper extremity weakness, numbness, and slurred speech, in mid-2019. She was seen at the emergency department when the symptoms first started, and the CT head was negative for acute abnormalities. She was discharged to home with instructions to follow up with her primary care provider (PCP), as her symptoms spontaneously resolved in the ED. Two days later, she started having recurrent left-hand weakness lasting 10-15 minutes. Mild headache and lightheadedness was associated with weakness. No visual disturbances, facial droop, word-finding difficulties, lower extremity symptoms, or gait abnormalities were noted. She was admitted for further workup.

**Figure 1:**
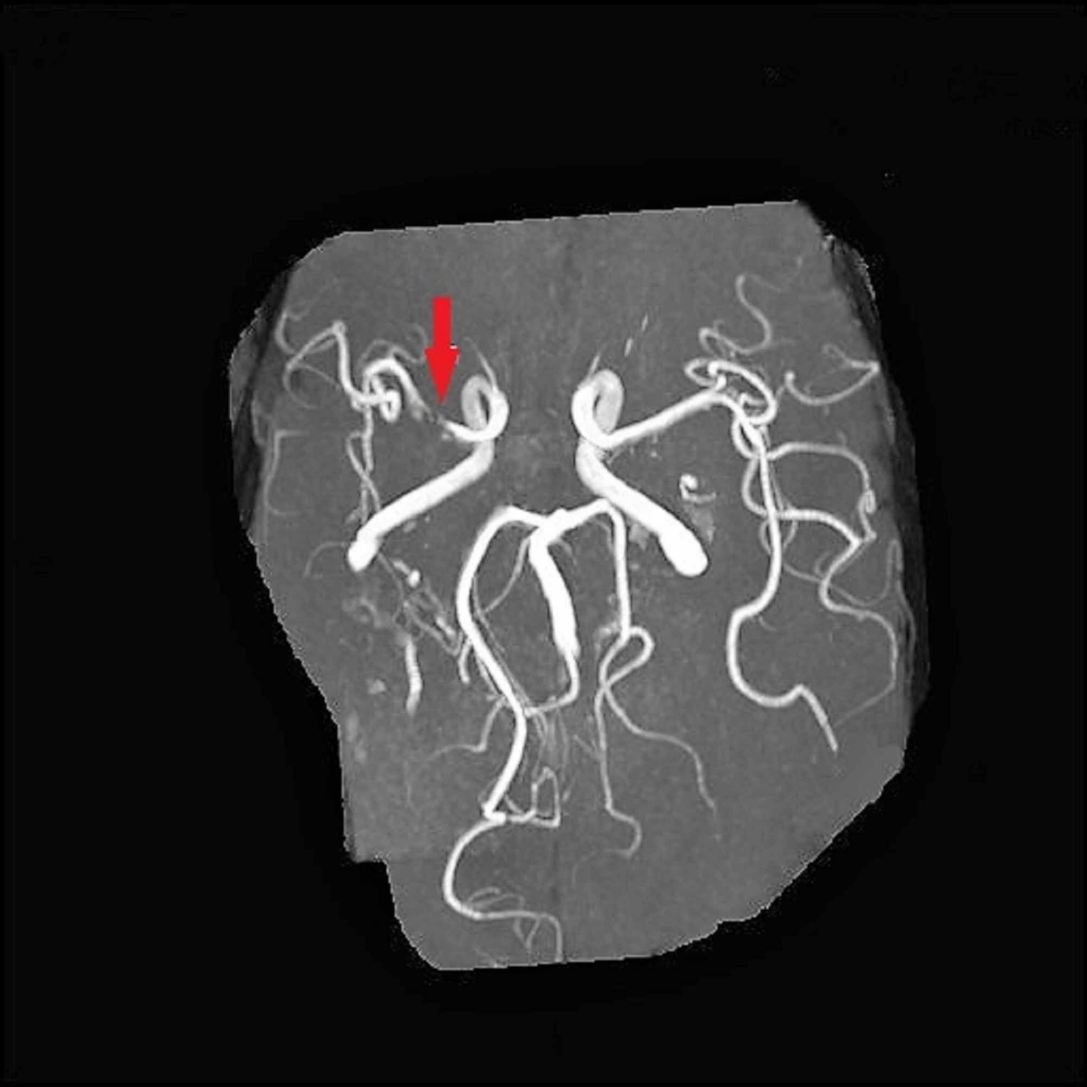
The magnetic resonance angiogram of the head shows a high-grade stenosis versus segmental occlusion of the right middle cerebral artery M1 segment (red arrow)

The magnetic resonance angiogram of the head revealed right hemisphere infarcts, bilateral anterior cerebral artery occlusions, high-grade stenosis versus segmental occlusion of the right middle cerebral artery M1 segment, and tiny collateral vessels adjacent to the right middle cerebral artery and bilateral posterior cerebral arteries raising the question of Moyamoya disease (Figure 1).

**Figure 2:**
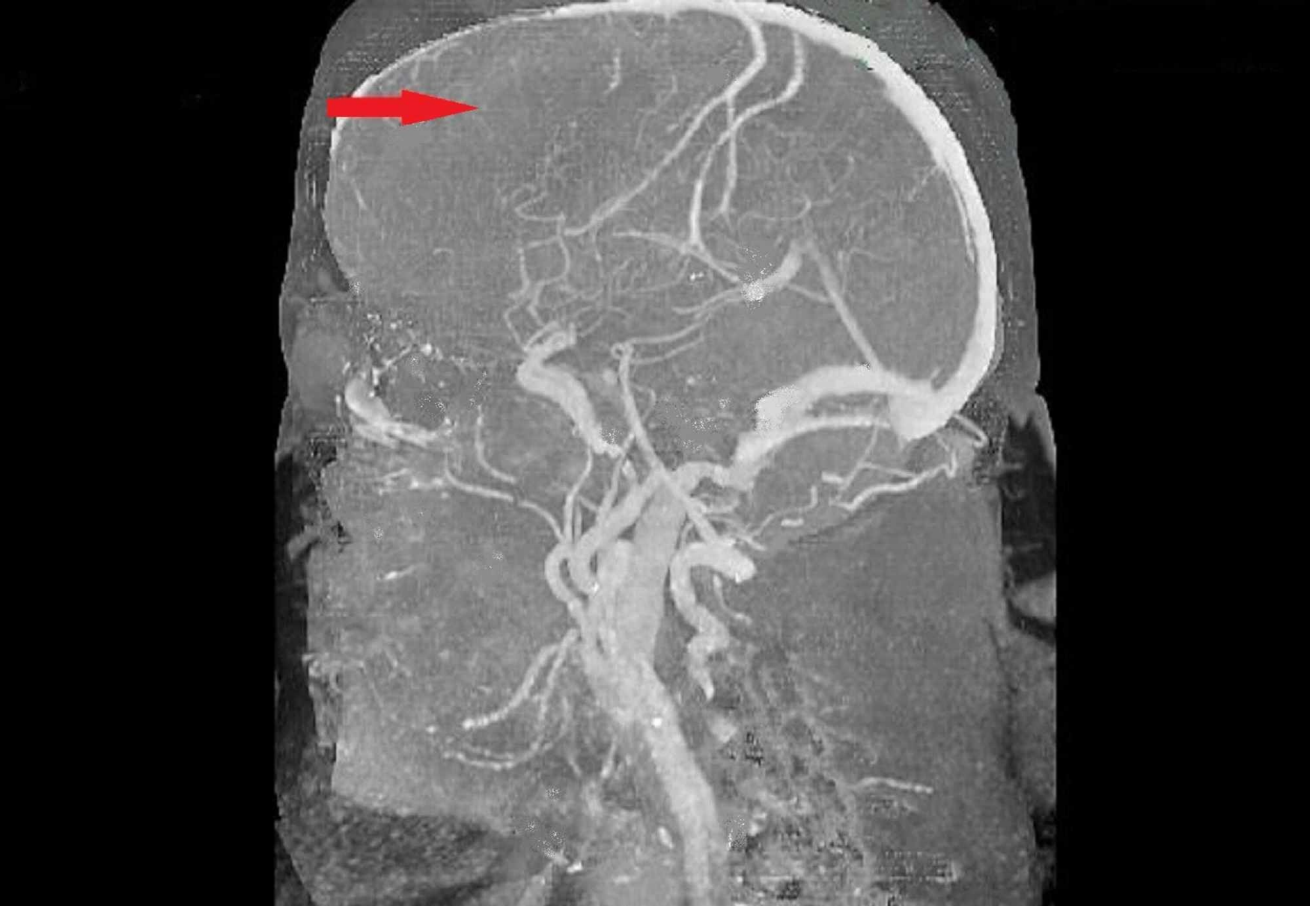
Computed tomography angiography shows occlusion of the bilateral anterior cerebral arteries (red arrow)

Computed tomography angiography was performed, and it showed occlusion of the bilateral anterior cerebral arteries. It also demonstrated possible tiny collateral vessels adjacent to the right middle cerebral artery and anterior cerebral arteries, which continued to raise suspicion of Moyamoya. The patient was then advised to undergo diagnostic catheter angiography (Figure 2).

**Figure 3:**
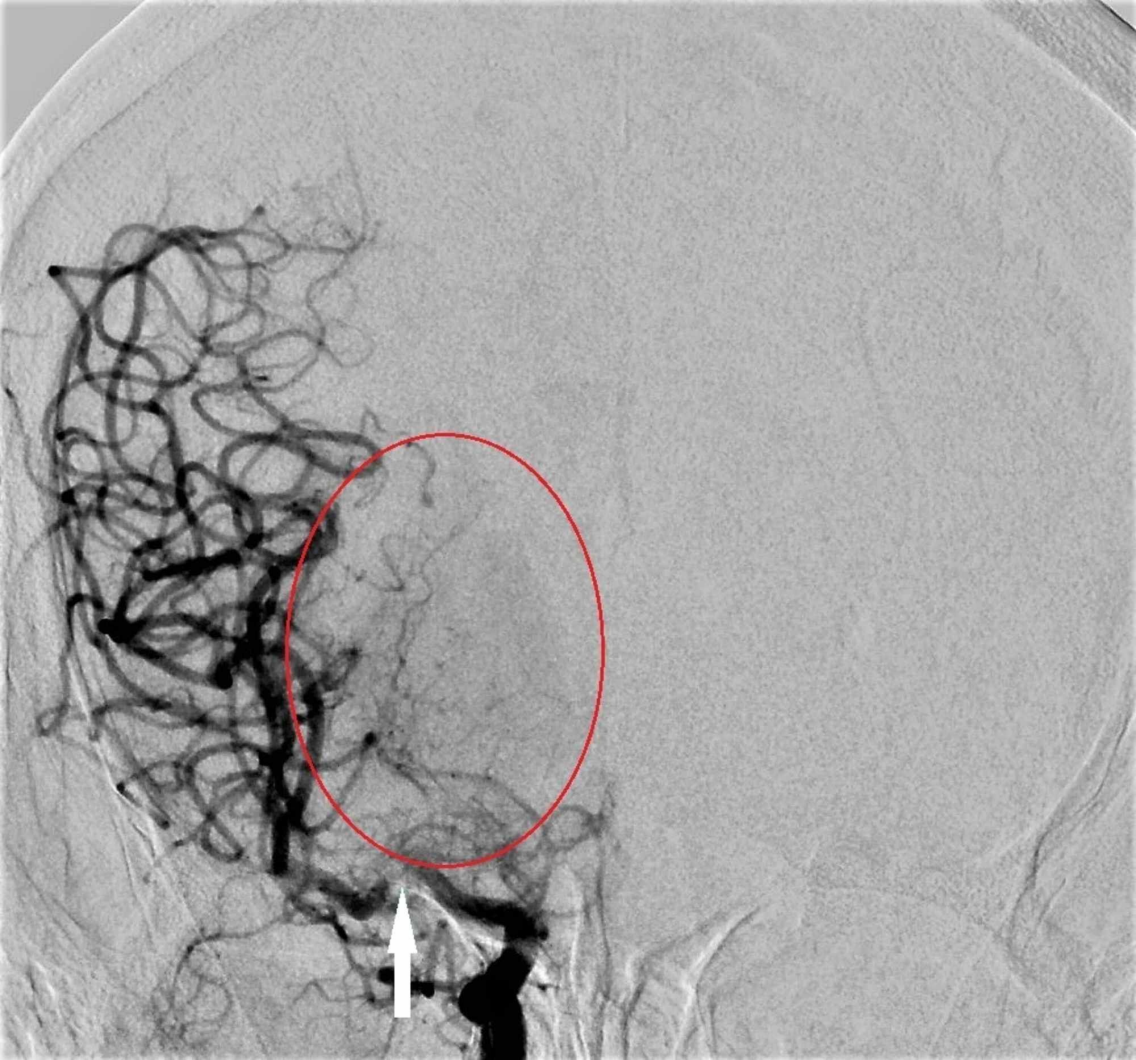
Diagnostic cerebral angiogram shows a high-grade stenosis involving the right middle cerebral artery (white arrow). A ridge network of right-sided collateral vessels consistent with a Moyamoya appearance is noted (red circle)

Diagnostic cerebral angiogram was completed, that showed a high-grade stenosis involving the right middle cerebral artery. Additionally, there was a ridge network of right-sided collateral vessels consistent with a Moyamoya appearance. She was started on Aspirin, Plavix, and atorvastatin therapy (Figure 3).

A hypercoagulation profile was ordered. She was found to be negative for Factor 5 Leiden, negative for prothrombin gene mutation, and negative antinuclear antibody. IgM and IgG for anticardiolipin were negative, and so was IgA, IgM, and IgG for beta glycoprotein.

She was evaluated by a physical therapist, occupational therapist, and speech therapist, and was cleared to return home without further therapy indicated. The National Institutes of Health Stroke Scale (NIHSS), a systematic assessment tool that provides a quantitative measure of stroke-related neurologic deficit, was found to be Zero.

The patient had an appointment with a neurosurgeon following discharge who discussed the pros and cons of the three different management approaches at length, namely, medical therapy, endovascular revascularization, and open bypass superficial temporal artery to the middle cerebral artery (STA-MCA). After mutual decision making, she chose medical therapy with dual antiplatelets and postponed invasive intervention until another event.

The neurosurgeon decided that clinical surveillance with phone calls every three months will take place and she will schedule in-person visits at six months. Red flags were discussed with the patient and she was advised to seek immediate medical attention if she developed any of them, and she agreed with the treatment and plan. The patient has been compliant with her office visits and is currently asymptomatic and completely functional at baseline. As of early June, when she was last seen in the office, she denied any new neurological symptoms and is presently on dual antiplatelet therapy, including Aspirin and Plavix.

## Discussion

Incidence/Prevalence

Moyamoya disease (MMD) is a chronic occlusive cerebrovascular condition with an unknown etiology. However, recent genetic studies have identified RNF213 in the 17q25-ter region as an important gene involved in the disease among East Asian populations [2]. Due to genetic differences, moyamoya was initially considered more prevalent in people of East Asian ethnicity and specifically in Japanese, than people in the Western Hemisphere [2]. It is the most common pediatric cerebrovascular disease in Japan, with about three cases per 100,000 children. Women are nearly twice as affected as males [3]. The condition is now being reported all over the world and in people of all ethnicities.

Signs and symptoms

Symptoms of moyamoya are attributed to changes in the blood flow resulting from chronic progressive narrowing of the arteries at the base of the brain. The symptoms are categorized into two major groups: brain ischemia (stroke, TIA and seizures) and symptoms resulting from compensatory mechanisms responding to ischemia such as hemorrhage and headache from dilated collateral vessels [3]. Ischemic symptoms are seen in both children and adults, and intracranial hemorrhages are more commonly seen in adults than in children [2]. The most common symptoms of ischemia moyamoya are motor disturbances such as chorea, choreoathetosis, dyskinesia, dystonia, limb-shaking, and epilepsy [4]. In hemorrhagic moyamoya, the most common symptom is consciousness disturbance. Headaches are seen in 20% of those affected with moyamoya and are vascular in origin with features similar to migraines and may cause impairment of activities of daily living [4].

Diagnosis

Various invasive and semi-invasive studies can be used to identify MMD [5]. Transcranial Doppler can be used bedside to detect stenosis, but CT or MRI can detect ischemic vs. hemorrhagic strokes. CT angiography is the semi-invasive technique that can help classify the disease into Suzuki stages 1-6 based on the angiographic progression. While original diagnostic criteria required bilateral internal carotid artery (ICA) steno-occlusive changes, it was revised in 2015 to include unilateral and bilateral ICA stenosis leading to compensatory abnormal vasculature at the base of the brain [6]. Diagnostic criteria also specified the requirement of catheter angiography for unilateral cases and atherosclerotic changes, while MR imaging or catheter angiography can be used to diagnose bilateral cases.

Pathophysiology

Genetic and environmental factors have been conceptualized to play a role in the pathogenesis of the disease [2]. With 10% of the disease being familial, the strongest genetic association has been with the Ring factor 213 (RNF213) gene. Circulating endothelial progenitor cells, mainly responsible for neovascularization in postnatal physiology, have been known to increase in patients with MMD and decrease after revascularization procedure [7]. RNF213 was also associated with increased serum microRNAs that have multiple functions, including angiogenesis. Blood vessel changes of intimal proliferation include constrictive remodeling (outer diameter of major arteries is narrowed early in disease) and concentric enhancement in affected segments [8].

Management

MMD is a disease with progression rate in the natural clinical course of about 20% in six years, with female sex being an independent risk factor for progression [9]. Symptomatic treatment is indicated in acute ischemic or hemorrhagic stroke presentations, including decreasing intracranial pressure, seizure prophylaxis, draining intraventricular hemorrhage, and thermoregulation [5]. Current literature defines surgery as the mainstay treatment for secondary prophylaxis. Three types of surgical interventions have been identified - direct (branches of external and internal carotid are connected after bypassing stenosed arteries), indirect (brain surface is fed by angiogenesis due to direct contact with surgically placed vascularized tissue supplied by external carotid artery) and combined. Antiplatelets (e.g., Aspirin) can be used for prophylaxis in asymptomatic or hemodynamically stable individuals. However, none of the interventions have shown improved outcomes [5].

Prognosis

The long-term prognosis in asymptomatic patients with moyamoya is unclear. In symptomatic moyamoya, retrospective trials show that in order to prevent stroke, bypass surgery is more effective than conservative treatment [10]. In order to reduce ischemic and hemorrhagic stroke rate and improve neurological and neuropsychological outcomes, revascularization surgery plays a vital role [11]. Many complications can arise from angioplasty and/or stent placement including intracerebral hemorrhage and inability to effectively dilate the treated vessels. There is no evidence that angioplasty or stenting improves the natural history of moyamoya and both are associated with early clinical and angiographic recurrence of symptoms [12].

## Conclusions

This case highlights the fact that Moyamoya disease can have an elusive presentation. Clinicians should have a keen eye and consider Moyamoya disease as a differential diagnosis in a patient presenting with symptoms of cerebrovascular accident. More research is needed to have a better understanding of the natural history of the disease as well as its management.
